# Reflexive anaphor resolution in spoken language comprehension: structural constraints and beyond

**DOI:** 10.3389/fpsyg.2014.00904

**Published:** 2014-08-19

**Authors:** Kaili Clackson, Vera Heyer

**Affiliations:** ^1^Department of Language and Linguistics, University of EssexColchester, UK; ^2^Potsdam Research Institute for Multilingualism, University of PotsdamPotsdam, Germany

**Keywords:** binding principle A, reflexive resolution, discourse prominence, sentence processing, eye-tracking

## Abstract

We report results from an eye-tracking during listening study examining English-speaking adults’ online processing of reflexive pronouns, and specifically whether the search for an antecedent is restricted to syntactically appropriate positions. Participants listened to a short story where the recipient of an object was introduced with a reflexive, and were asked to identify the object recipient as quickly as possible. This allowed for the recording of participants’ oﬄine interpretation of the reflexive, response times, and eye movements on hearing the reflexive. Whilst our oﬄine results show that the ultimate interpretation for reflexives was constrained by binding principles, the response time, and eye-movement data revealed that during processing participants were temporarily distracted by a structurally inappropriate competitor antecedent when this was prominent in the discourse. These results indicate that in addition to binding principles, online referential decisions are also affected by discourse-level information.

## INTRODUCTION

According to most theoretical accounts, the interpretation of a reflexive is determined solely by a structural constraint which identifies a unique referent (Chomsky, 1981, 1986; Levinson, 1987; Pollard and Sag, 1992; Reinhart and Reuland, 1993; Reinhart, 2000, Reuland, 2001; Burkhardt, 2005 among others). For example, Principle A requires that an English argument reflexive is bound by a local antecedent that falls within its governing category, so that the anaphor and its antecedent are co-indexed (i.e., have compatible number, gender and person features), and the anaphor is c-commanded by its antecedent. In (1) *Susan* is structurally accessible as an antecedent as *Susan* binds (i.e., c-commands and is co-indexed with) *herself* and falls within the governing category of *herself* (shown by square brackets). *Jane* falls outside the governing category of *herself* and so is not structurally accessible as an antecedent.

(1) Jane_1_ says that [Susan*_2_* hurt herself*_∗1/2_*].

In recent years there has been considerable discussion about the role that such structural constraints play in online sentence processing. Of particular interest is whether the parser’s search for a referent is guided principally by structural considerations, where each potential antecedent is assessed based on its structural position; or whether a more cue-based search is implemented, where a structurally illicit referent that is strongly supported by other cues (such as being of appropriate gender and number, and in a prominent position) might be briefly considered and so lead to interference effects [for further discussion see Van Dyke (2007), Phillips et al. (2010), and Dillon et al. (2013) among others]. As the referent for a reflexive can be identified on the basis of structural information alone (in contrast to pronouns where structural information rules out certain referents, but does not necessarily identify a single referent), reflexive resolution is often seen as a good test case in this debate. In the present study we ask whether a noun phrase in a position where co-reference with the reflexive would violate a constraint, henceforth termed “inaccessible,” [such as *Jane* in (1)] is ever considered by the parser as a potential referent. Results from previous research have pointed to somewhat differing conclusions, leaving this question unresolved.

For example, early cross-modal priming studies (Nicol, 1988; Nicol and Swinney, 1989) suggested that during reflexive resolution, the structural constraint acts as an early filter so that the adult parser only considers structurally accessible antecedents but not structurally inaccessible ones^1^. Evidence to support this has also come from studies using more time-sensitive measures such as ERPs and eye-tracking during listening (Xiang et al., 2009; Clackson et al., 2011) where no effects of the inaccessible antecedent were found^2^. In contrast, using a self-paced reading task Badecker and Straub (2002) found that reading times on the second word following the reflexive were significantly longer when the gender of the inaccessible antecedent matched that of the reflexive compared to when it did not, suggesting that the parser briefly considered the inaccessible antecedent as a potential antecedent. Furthermore, although results from eye-tracking during reading experiments are somewhat mixed, a number of studies have found tentative evidence that the inaccessible antecedent is not fully ruled out by Principle A. For example, Cunnings and Felser (2013) found that the gender of the inaccessible antecedent affected reading times both at the reflexive region and text downstream of the reflexive, while Sturt (2003) found an effect in second-pass reading times on the reflexive and later regions^3^. While a number of studies have not found evidence of interference effects (e.g., Felser et al., 2009; Dillon et al., 2013) it is possible that such null results are due to particular properties of the materials used (see Discussion section), or stem from a lack of power to detect a relatively small effect [see Chen et al. (2012) for further discussion on power].

One difficulty in interpreting previous results is that it is not certain whether participants interpreted the reflexive correctly. If previous studies included comprehension questions, they were usually not aimed at the interpretation of the critical reflexive in order to avoid drawing participants’ attention to the purpose of the experiment. Therefore, in most experimental paradigms there is no oﬄine measure of the interpretation of the reflexive, making it impossible to know whether the observed results reflect successful processing of the reflexive or not. Indeed, one oﬄine study showed that participants incorrectly interpreted a reflexive as referring to a gender matching but structurally inaccessible antecedent in 17% of cases (Sturt, 2003). Furthermore, a number of the studies above rely on gender stereotype nouns (such as *surgeon* being assumed to be male) to create “gender match” and “gender mismatch” conditions, and again it is impossible to know if participants interpreted such nouns in the manner intended.

The present eye-tracking during listening study avoids such difficulties by only using proper names for potential antecedents and by using a “goal-directed” design. The advantage of such a design is that the participant is required to identify the referent for the reflexive for each trial, thus allowing for separate analysis of eye movements and response times for trials where participants did, and did not, interpret the reflexive correctly. Trueswell (2008) supports such designs, arguing that eye movements reflect “goal-directed behavior” and that it is only possible to infer referential decisions from eye movements when these decisions are necessary to achieve the task at hand. The “goal-directed” design was chosen because a naturalistic design, with participants simply looking at pictures while listening to auditory stimuli, can lead to less data relevant to the research question due to participants not paying attention to the pictures at critical points. For instance, Clackson et al. (2011) investigated reflexive resolution using eye-tracking during listening by asking participants to listen to stimuli and answer general comprehension questions which did not probe the referent of the reflexive. One effect of this naturalistic task was that participants’ attention was in no way drawn to the non-salient reflexive. As a result, in approximately half the trials participants did not look at any potential antecedent on hearing the reflexive, considerably reducing the quantity of relevant eye movement data collected. Therefore, it is possible that the observed numerical trend showing an effect of the inaccessible antecedent soon after hearing the reflexive (i.e., fewer looks to the accessible antecedent and more looks to the inaccessible antecedent when the inaccessible antecedent matched in gender with the reflexive) did not turn out to be statistically reliable due to the limited data collected.

In the present study the participants’ task was presented as a “Who is it for?” activity where participants were asked to identify as quickly as possible which character in a story received a particular object. In experimental trials the recipient was identified by a reflexive. Gaze direction across a scene which included the participants in the story was monitored, so that three responses were recorded: accuracy of identifying the recipient character, response time, and gaze direction at the point of the crucial reflexive. If manipulation of the gender of the inaccessible antecedent (matching or mismatching the gender of the reflexive) affects responses, this interference effect would suggest that the inaccessible antecedent was briefly considered as a potential antecedent in the early stages of processing.

## MATERIALS AND METHODS

### PARTICIPANTS

Forty-two native speakers of English (mean age: 23, range: 18–48, 16 males) were recruited at the University of Essex and were paid for their participation. All participants had normal or corrected-to-normal vision.

### DESIGN AND MATERIALS

The auditory materials were taken from the reflexive conditions used by Clackson et al. (2011) consisting of spoken pairs of sentences, each involving two characters from the set of Susan, Peter, Mr. Jones, and Mrs. White. The first sentence introduced the first character and established a suitable context for the second sentence, which included the second character, an inanimate object, and the critical reflexive. In each trial, the object was for, or was given to, the second character (the recipient), referred to by a reflexive. The auditory stimulus set comprised 24 experimental items, each appearing in two conditions. In the Double-Match condition the gender of both characters matched that of the reflexive, and in the Single-Match condition only the gender of the accessible antecedent matched that of the reflexive, as illustrated in (2).

(2) *Double-Match*
Peter was waiting outside the corner shop. He watched as Mr. Jones bought a huge box of popcorn for himself over the counter.
Single-Match
Susan was waiting outside the corner shop. She watched as Mr. Jones bought a huge box of popcorn for himself over the counter.

The inaccessible antecedent [*Peter* or *Susan* in (2)] is in a discourse prominent position as it is the first-mentioned character and the subject of both main clauses (repeated as a pronoun in the second one). The accessible antecedent (here: *Mr. Jones*), in contrast, is less salient as the subject of the subordinate clause.

Auditory stimuli were recorded using splicing to ensure that each version of an item was identical except for the name and pronoun changes necessary for the experimental manipulation.

Experimental items from a separate pronoun experiment were presented together with those from the present reflexive study, so that in addition to the reflexive experimental trials, each participant heard 24 pronoun items which mirrored the structure of the reflexive items, and 48 filler trials comprising a range of different grammatical constructions and featuring some additional characters (Doctor, Nurse, King, and Queen). Filler trials were similar to the experimental items in that the recipient of an object was introduced by a preposition (*for*, *to*, *on,* or *at*), but other properties were manipulated to provide variety of structure: the number of characters introduced before the preposition varied from one to three and, in contrast to the experimental items, the majority of filler items identified the recipient by name. This meant that contexts in which the recipient was only introduced after the preposition could be created, thus preventing participants from assuming that the recipient would always be mentioned early in the sentence. Furthermore, the point at which it became obvious which character received the object was varied in the filler items so that participants did not know when to expect the information which provided the answer to the task. For example, the recipient of the object is mentioned quite early in (3) but fairly late in (4) (object is underlined and recipient is shown in bold).

(3) At the hospital the nurse got a glass of water for **the doctor** because he had bad hiccoughs and needed to see a patient.
(4) After the accident in the royal carriage the King and the Queen were very upset. The doctor visited them and put a plaster on **the Queen’s** nose where she had cut it.

**FIGURE 1:**
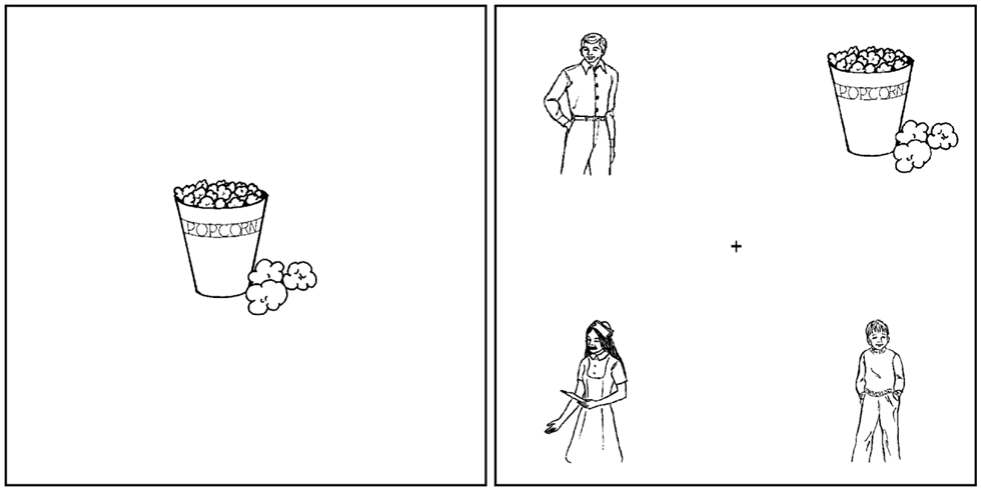
**Example visual displays for auditory stimuli shown in (2) (Double-Match condition)**.

Each auditory trial was accompanied by two visual displays as shown in **Figure 1**. A picture of the inanimate object was shown in the centre of the screen prior to the start of the auditory stimulus, and this was followed by the main visual display comprising four pictures: the inanimate object and three animate characters, which was viewed while the auditory stimulus was heard. For experimental trials, two of these characters were mentioned in the auditory stimulus and one (mismatching the gender of the reflexive) served as a distracter.

The four pictures were positioned in the corners of the screen, with a small cross in the center, and the positioning of the pictures of the characters and the inanimate object was counterbalanced across items. All pictures were black-and-white line drawings, of approximately the same size, and were not noticeably different in terms of visual saliency. All pictures were selected from a set of 520 pictures from the International Picture Naming Project (http://crl.ucsd.edu/∼aszekely/ipnp/) for which various normed measures are available^4^. Experimental trials were arranged in four lists according to a Latin Square design (due to the similarity between the two reflexive conditions and two pronoun conditions from a separate experiment) so that each participant saw each trial in only one condition (Double-Match or Single-Match). The same set of filler trials was used with each list, and trials were presented in a pseudo-randomized order such that no more than two experimental trials occurred consecutively. To counteract any effects of fatigue, the four lists were then reversed to create eight lists in total so that items heard early in the experiment by one participant were heard late in the experiment by another. The study received ethical approval from the University of Essex ethics committee.

### PROCEDURE

Participants sat two meters away from a projection screen where the visual display measured 170 × 120 cm, and during the experiment their eye movements were recorded by a digital camcorder recording 25 frames per second (i.e., one frame every 40 ms) which was placed below the projection screen and trained on the participant’s face. This set-up ensured that when the video was played back, participants’ eye movements between pictures were distinct enough to be clearly interpreted. The presentation of visual and auditory stimuli was programmed using DMDX (Forster and Forster, 2003), and the sound output from the computer was split, going directly to both the headphones worn by the participant, and to the video camera so that the sound recorded by the video camera was exactly synchronized with what the participant heard. Participants were provided with full details of the procedure and gave written consent before the testing session started.

At the start of each trial, a cross appeared on screen for 1 second, followed by a picture of the object mentioned in the story, which remained in the centre of the screen for 3 seconds. The participant’s task was to play a game of “Who is it for?,” identifying the recipient of this object while listening to the story which followed. Following the picture of the object, the main visual display for that item was shown on screen for 1 seconds before the auditory stimulus began, and remained on screen until the next trial began. Participants were asked to listen carefully to the story and respond as quickly as possible once they knew who the object was for, by pressing the button on the gamepad which corresponded with the position of the selected character on the screen. For example, if the recipient was identified as being the character in the top left quadrant of the screen, the participant would press the top left button. If participants answered incorrectly the word “OOPS!” was displayed on the screen to encourage participants to pay closer attention and to discourage hasty responses before the recipient had been identified in the story. There was no feedback for correct responses. The next trial was initiated automatically, independent of the participant’s response. Participants were introduced to all the characters and their pictures at the start of the session, and in order to get used to the pictures and the process of selecting the recipient of the object on the gamepad, the experiment was preceded by six practice trials. For these trials the stories were presented over loudspeakers to allow for immediate questions by the participant as well as to enable the experimenter to check that participants responded shortly after the key word and did not wait until the end of the story. If a participant was not completely confident with the procedure after this, the practice session was repeated. During the main experiment, participants listened to stimuli through headphones and were offered three breaks, one after every 18 items. The entire session took approximately 35 minutes.

Three dependent measures were taken and analyzed: response accuracy (the accuracy with which participants correctly interpreted the reflexive to identify the recipient of the object), response times, and eye movements. For statistical analyses, response accuracy was recorded as either correct or incorrect. Reaction times were calculated as the delay between the onset of the reflexive and when the response button was pressed. Video footage of participants’ eye movements was analyzed using ELAN annotation software (Brugman and Russel, 2004), and gaze direction was recorded every frame for 2000 ms (50 frames in total) from the onset of the critical reflexive. The still image for each frame (every 40 ms), was inspected to determine the direction of gaze (toward one of the four pictures, the center of the screen or off-screen), and a target was counted as “fixated” for every frame where eyes were directed toward that picture^5^. Off-screen looks (which accounted for 2.2% of the total dataset) were treated as missing data.

## RESULTS

All analyses were carried out on raw data using mixed-effects regression modeling in “R,” version 3.0.1 (Baayen et al., 2008; R Development Core Team, 2010). Models included participant and item random effects, and to account for the fact that gaze direction in consecutive frames is not independent (gaze direction in any particular frame is heavily influenced by gaze direction in the previous frame), random effects of Trial were also included for analyses of eye movement data. Maximal random effects structure was used so that as well as random intercepts, all fixed effects and interaction terms had corresponding random slopes by participant, item, and trial as appropriate (Barr et al., 2013). Best fitting models were identified by adding predictors incrementally to an empty model, with those that resulted in a significant improvement of the fit of the model being retained. In the analysis of eye movements, the fixed factor of Time was added to the model in order to test for differences between conditions over time (i.e., proportions of looks increasing or decreasing differently across the two conditions). Due to the non-linear relationship between looks and Time, second and third order polynomials of Time were also tested as predictors. The response accuracy and eye movement data were analyzed using logistic regression due to the categorical nature of the data. For eye movement data the binary dependent variable encoded whether the picture of a particular antecedent was, or was not, fixated for each of the 40 ms frames. Tables/graphs show grand mean results as participant and item differences are accounted for in the mixed-effects analysis.

As the oﬄine measure allows for the identification of trials in which the final interpretation of the reflexive was incorrect, and as response times and eye movements in trials where the inaccessible antecedent (or another incorrect answer) was selected do not reflect successful processing, incorrectly answered trials (comprising 3.6% of the total data set) were not included in the analysis of response times or eye movements.

### RESPONSE ACCURACY

As shown in **Table 1**, response accuracy was high (above 95%) in both conditions. In the Double-Match condition the majority of errors were due to the selection of the inaccessible antecedent.

**Table 1:** Oﬄine button press responses.

		Correct responses	Incorrect responses	
		% Accessible antecedent	% Inaccessible antecedent	% Other erroneous responses
Double-Match		95.2	4.4	0.4
Single-Match		97.6	0.4	2.0

Analysis of accuracy scores (with each response coded as correct or incorrect) showed no effect of Condition (adding Condition as a fixed factor did not improve the fit of the model over an empty model).

### RESPONSE TIMES

**Table 2** shows the mean response times for correctly identified recipients. Participants took more time to identify the referent when both antecedents matched the reflexive in gender.

**Table 2:** Mean response times (and standard deviation) for correctly answered trials.

	Response time
Double-Match	1155 (688)
Single-Match	1043 (687)

Statistical analyses confirmed that response times were significantly longer in the Double-Match condition [Condition (Double-Match): β = 101.28, SE = 44.83, *t* = 2.259].

### EYE MOVEMENTS

**FIGURE 2:**
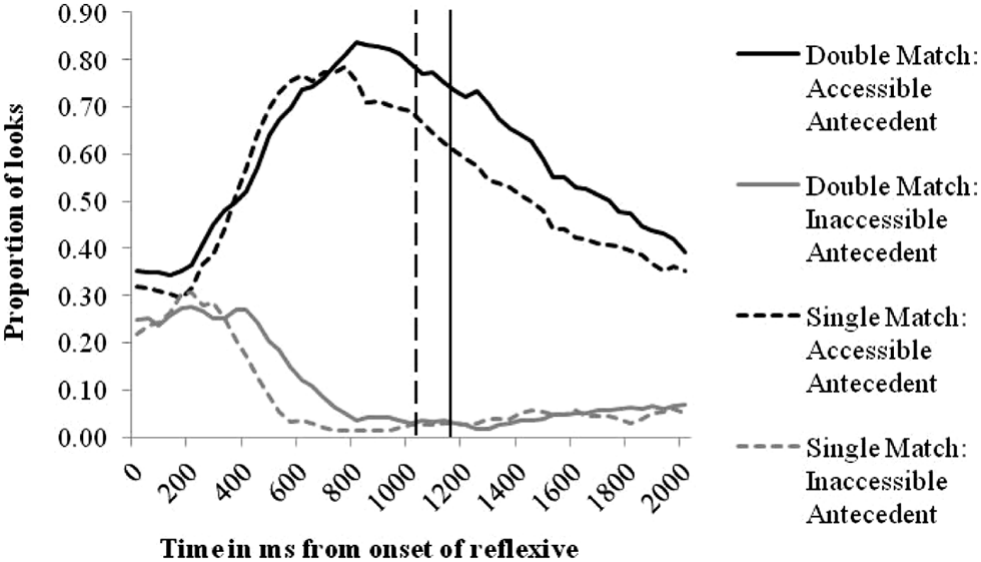
**Proportions of looks to potential antecedents**.

**Figure 2** shows fixations of the two potential antecedents in the two experimental conditions (Double-Match/Single-Match) during the 2 second following the onset of the critical reflexive. The *x*-axis displays the time in milliseconds from the onset of the reflexive, and the *y*-axis depicts the proportions of looks to the two potential antecedents, i.e., the number of trials in which a participant fixated on a particular picture for each 40 ms video frame as a proportion of the total number of trials in which they were looking at the screen. As it takes approximately 200 ms to program an eye movement (Rayner et al., 1983), only changes in proportions of looks after 200 ms can be attributed to participants hearing the reflexive. Note that while the graph shows grand mean data plotted on a proportional scale for ease of interpretation, the statistical analysis uses a logistic scale (as analysing data on a proportional scale can lead to inaccurate estimation of effects) and takes into account the clustering of data for each participant, item, and trial.

From 200 ms after hearing the reflexive, the proportion of looks to the accessible antecedent (black lines) increases sharply in both conditions, and looks to the inaccessible antecedent (gray lines) fall. The vertical lines in **Figure 2** indicate the mean response time for each condition (solid line = Double-Match, broken line = Single-Match). Proportions of looks to the other areas of the screen not shown in the graph (object picture, distracter picture and center of the screen) were low throughout the time window (typically between 0 and 0.15), with looks to the object gradually increasing to 0.30 after 1200 ms. The proportion of looks to each of these screen areas was similar across conditions, but slightly higher in the Single-Match condition than the Double-Match condition.

In order to investigate the time course of effects, in the statistical analysis models were fit to 400 ms time windows (200–600 ms, 600–1000 ms, 1000–1400 ms, and 1400–1800 ms). These time windows were selected following visual inspection of the data.

It is important to note that differences between conditions may be seen in two different ways: it may be that in any particular time window the average proportion of looks to an antecedent is higher in one condition than another, or it may be that the rate of increase/decrease in looks (shown by the slope or curve) differs. To investigate the first possibility, models were fit to test for an interaction between Antecedent (Inaccessible/Accessible) and Condition (Single-Match/Double-Match). To explore the second possibility, models also tested for an interaction between Antecedent, Condition, and Time. Thus findings of an Antecedent × Condition interaction, or an Antecedent × Condition × Time interaction each signify (in slightly different ways) that participant performed differently across the two conditions. In later discussion of results, the general term *effect of the inaccessible antecedent* will be used to cover both types of effect.

As shown in **Table 3**, statistical analyses revealed significant interactions between Antecedent, Condition, and Time, in the 200–600 ms and 600–1000 ms time windows. These results show that gaze direction was affected by the gender of the inaccessible antecedent until at least 1 second after the onset of the reflexive.

**Table 3:** Antecedent × Condition and Antecedent × Condition × Time interactions from best fitting models (full results are shown in Appendix A, found in the Supplementary Material).

Time window (ms)	Fixed effects	β	SE	*z* value	*p* value
200–600	Ant (Inacc.) × Condition (Double-Match)	6.694	5.152	1.299	0.194
	Time × Ant (Inacc.) × Condition (Double-Match)	31.792	10.455	3.041	0.002*
600–1000	Ant (Inacc.) × Condition (Double-Match)	-3.256	4.124	-0.790	0.430
	Time × Ant (Inacc.) × Condition (Double-Match)	-39.849	18.122	-2.199	0.028*
1000–1400	Ant (Inacc.) × Condition (Double-Match)	-5.412	8.575	-0.631	0.528
	Time × Ant (Inacc.) × Condition (Double-Match)	-39.850	22.241	-1.792	0.073
1400–1800	Ant (Inacc.) × Condition (Double-Match)	-24.920	34.553	-0.721	0.471
	Time × Ant (Inacc.) × Condition (Double-Match)	84.467	60.952	1.386	0.166

*p < 0.05.

In order to further investigate the source of the interactions, looks to each antecedent were analyzed separately for the 200–600 ms and 600–1000 ms time windows, as shown in **Table 4**.

**Table 4:** Main effect of Condition and Time × Condition interactions from best fitting models fit to looks to each antecedent.

Time window (ms)	Fixed effects	β	SE	*z* value	*p* value
200–600	**Looks to accessible antecedent**
	Time	59.051	8.840	6.680	<.001*
	Condition (Double-Match)	0.321	2.072	0.155	0.877
	Time × Condition (Double-Match)	-19.099	6.948	-2.749	0.006*
	**Looks to inaccessible antecedent**				
	Time	-302.20	124.53	-2.430	0.015*
	Condition (Double-Match)	18.460	29.21	0.632	0.527
	Time × Condition (Double-Match)	83.520	143.15	0.583	0.560

600–1000	**Looks to accessible antecedent**
	Time	-1.120	13.919	-0.080	0.936
	condition (Double-Match)	4.959	4.689	1.058	0.290
	Time × Condition (Double-Match)	40.503	18.646	2.172	0.030*
	**Looks to inaccessible antecedent**				
	Time	-3.211	20.726	-0.155	0.877
	Condition (Double-Match)	-4.496	5.598	-0.803	0.422
	Time × Condition (Double-Match)	-34.00	28.025	-1.213	0.225

*p < 0.05.

From 200 to 600 ms looks to the accessible antecedent increased more slowly in the Double-Match condition than in the Single-Match (shown by the negative slope for the Time × Condition interaction), while, in contrast, from 600 to 1000 ms there was a greater increase in looks to the accessible antecedent in the Double-Match condition (shown by the positive slope for the Time × Condition interaction). While the lack of significant effects in the looks to the inaccessible antecedent shows that there is not a direct relationship between looks to the two antecedents (i.e., a lower proportion of looks to the accessible antecedent does not directly correspond with an increase in looks to the inaccessible antecedent – recall that gaze was distributed over five screen regions), it is nevertheless the case that the presence of a gender matching inaccessible antecedent leads to slower initial identification of the correct antecedent, and then to prolonged looking at the accessible antecedent prior to giving a response to identify the recipient.

### SUMMARY OF RESULTS

While oﬄine accuracy in determining the referent for the reflexive was not affected by the gender of the inaccessible antecedent, response times were significantly longer when the gender of the inaccessible antecedent matched that of the reflexive (Double-Match condition).

The analysis of eye movements also showed that the gender of the inaccessible antecedent significantly affected looks to the accessible antecedent over the first 1000 ms following the onset of the reflexive. When a gender matching competitor was present (i.e., in the Double-Match condition) participants were initially slower to identify the correct antecedent (200–600 ms), and then more likely to look at the correct antecedent as they prepared to respond to the task (600–1000 ms).

## DISCUSSION

Results showed that adults are significantly distracted by a gender matching but structurally inaccessible competitor antecedent. Eye movement data revealed a two-phase pattern, with early interference effects leading to faster identification of the accessible antecedent in the Single-Match condition, and a later effect whereby participants looked more at the accessible antecedent in the Double-Match condition.

One advantage of eye-tracking during listening over reading-based measures is the ability to focus more precisely on the nature of the effect. While reading-based measures can tell us whether the presence of a gender matching inaccessible antecedent has an effect on the processing of the reflexive, eye-tracking during listening experiments allow us to investigate the origin of that effect more precisely. In this case, we have seen not only that the gender of the inaccessible antecedent has an effect, but specifically that it affects looks to the accessible antecedent. This leads to two possible interpretations of our findings^6^. Firstly, it may be (as is traditionally assumed by studies finding effects of the inaccessible antecedent) that the gender-matching inaccessible antecedent is briefly considered as a potential referent by the parser, before being discarded on the grounds of structural position. If this were the case, one might expect significant effects in the looks to both the inaccessible antecedent and the accessible antecedent (more looks to the inaccessible and fewer to the accessible antecedent). Alternatively, it may be that a gender matching inaccessible antecedent has the effect of slowing down identification of the accessible antecedent, but is not specifically considered as an antecedent itself. Since it is not clear why the gender of the inaccessible antecedent should affect processing of the reflexive unless the inaccessible antecedent were being considered as a competitor, and bearing in mind oﬄine results showing that a gender matching inaccessible antecedent is frequently incorrectly interpreted as the referent for a reflexive (Sturt, 2003), we are inclined to support the former interpretation (arguing that there is clearly a numerical, though non-significant, trend toward increased looks to the inaccessible antecedent in the Double-Match condition). However, we acknowledge that the latter interpretation is possible, and that future research probing this distinction is needed. Under either interpretation, it is clear that processing the reflexive involves accessing the inaccessible antecedent, thus arguing against theories which claim that the early application of structural constraints makes inaccessible antecedents “invisible” to the parser.

Our results differ from those reported by Clackson et al. (2011) who used the same materials as the present study but a naturalistic listening task and found no significant effects of the inaccessible antecedent. However, visual inspection of their results shows a numerical effect between 200 and 600 ms similar to the early effect observed here, with a slower increase in looks to the accessible antecedent, and increased looks to the inaccessible antecedent in the Double-Match condition. In order to make a direct comparison between the present study and Clackson et al.’s (2011), data from the latter was re-analyzed using the same analysis methods as presented here (400 ms time windows, maximal random effects structure and including random effects of Trial), however, results showed no significant effects of the inaccessible antecedent^7^. Nevertheless, since early differences between conditions were seen in both experiments (although not significant in Clackson et al., 2011), this suggests that this effect is task-independent, i.e., similar results found using naturalistic and goal-directed designs. In contrast, the later effect appears to be task-specific: in the goal-directed task where participants are aware that the right or wrong response depends on the correct interpretation of the reflexive, we see more looks to the accessible antecedent in the Double-Match condition from 600 to 1000 ms, whereas when participants are required only to listen to auditory stimuli with no emphasis put on processing the reflexive, no such later effect is seen.

The suggestion that later effects may be more affected by the participant’s task is supported by evidence from ERP experiments where early and late ERP components differ with regard to their susceptibility to experimental variations. Both the early left anterior negativity (ELAN; occurring around 100–300 ms) and the P600 (occurring around 600–1000 ms) are associated with syntactic violations, but while the early effect is not affected by changes to the task, the later effect has been shown to be dependent on task manipulations such as the expected frequency of syntactic violations (Hahne and Friederici, 1999) and the specific instructions given to participants (Hahne and Friederici, 2002). Such results have led to the suggestion that the early effect reflects highly automatic processes, while the later effect reflects processes that are under the participant’s strategic control. Friederici (2002) identifies the P600 component with a process of “reanalysis and repair.” Since our participants were more likely to look at the picture of the accessible antecedent in the more challenging Double-Match condition immediately prior to responding, this may reflect a similar process of overcoming any earlier confusion and “checking” the answer. Logically, such a checking process would be absent when the task did not require the participant to give a response identifying the referent of the reflexive.

The cross-task differences in results observed for studies using the same auditory stimuli highlight the importance of identifying and separating task-independent and task-related effects. In eye-tracking during listening studies, the naturalistic listening method avoids participants adopting behavioral strategies to complete the task (as there is no task), but leaves questions about whether participants actually processed the linguistic element under investigation, and if so, whether their interpretation was in fact correct. In contrast, the goal-directed method forces participants to process the required language and gives a clear indication of the participant’s interpretation, although the results may also reflect the conscious processes involved in attaining the goal. It is only by systematic comparison of results from experiments using the same materials but differing designs that the role of the task can be identified. More studies of this sort are needed to confirm which effects are truly task-independent, and in the case of eye-tracking during listening studies, to further explore how cross-condition differences between looks to the target and looks to the competitor might be interpreted.

It might be suggested that a potential explanation for the early effect is that in the Double-Match condition participants initially interpret the first syllable of “himself/herself” as the pronoun “him/her,” leading to early eye movements toward the gender matching non-local antecedent before participants hear “… self.” However, acoustic comparison of the first syllable of “himself/herself” and the pronouns “him/her” carried out by Clackson et al. (2011) showed that the unstressed syllable in the reflexive was significantly reduced in duration and intensity compared to the pronoun. While pronouns often occur in phonologically weak forms, in the materials used here any pronoun occurring in the position of the reflexive would naturally be pronounced as a strong form, making it unlikely that participants would interpret the weak first syllable of the reflexive as a pronoun.

As outlined in the introduction, results from previous experiments using different methodologies differ with regard to the existence and timing of interference effects. In particular, eye-tracking during reading studies have revealed conflicting patterns of results (even when the materials were very similar), and where interference effects are reported, these are usually in “later measures” corresponding with Sturt’s (2003) “defeasible filter” theory, which proposes that although the inaccessible antecedent is initially blocked by the syntactic constraint, the parser may consider it at a later point in processing. In contrast, the results from the current study suggest that the interference caused by the gender matching inaccessible antecedent occurred relatively early in processing. While this apparent timing difference is still to be fully explained, it may be related to differences between auditory and visual processing or the fact that the two methodologies measure very different things, making it questionable whether reading times on the reflexive and following words can be directly compared with the probability of looking at a particular referent. Another contributing factor may be that the low salience of the reflexive affects reading designs in the same way that it can lead to participants failing to look at a potential antecedent in naturalistic listening designs. Specifically, the null effects in early reading measures could be due to high skipping rates and the resulting smaller amount of data points, i.e., a lack of power to detect small effects. For instance, Felser and Cunnings (2012) and Cunnings and Felser (2013) report skipping rates in the reflexive region of 11.2–15.6%, considerably higher than in the spill-over region (5.1–8.2%), raising the possibility that the reported null effect in early measures is due to a lack of power.

Connected to skipping rates, a further potential explanation for a lack of consistent effects in reading studies is the preview benefit in written texts. While orally presented sentences are presented one phoneme after the other, readers can visually inspect several letters at a time, both in the fovea and the parafovea. The fact that the reading span in English generally extends 14–15 letters to the right of the fixation allows readers to “look ahead” in the sentence [for reviews of research on parafoveal processing see Rayner (1998) and Schotter et al. (2012)]. Therefore, it is likely that in reading studies participants processed the reflexive parafoveally before actually fixating on it. With spaces and length information being very salient, the distinction between English reflexives (6–10 letters) and pronouns (2–4 letters) can easily be made on the basis of this formal information available in the parafovea. This might provide participants with a “head-start,” reducing potential surprise effects which lead to longer reading times when a reflexive does not refer to the gender matching and discourse prominent, but structurally inaccessible, antecedent.

Even across methodological boundaries, it is clear that the discourse prominence of the inaccessible antecedent plays a role in determining the extent to which it can interfere with processing of the reflexive. In the present study and previous research reporting interference effects, the materials used were constructed such that the inaccessible antecedent was promoted in the discourse by being both in first-mentioned position and the matrix subject (Badecker and Straub, 2002; Sturt, 2003; Cunnings and Felser, 2013). In contrast, studies using materials where the inaccessible antecedent was not in first mentioned or matrix subject position (Xiang et al., 2009; Dillon et al., 2013), or where the prominence of the inaccessible antecedent relative to that of the accessible antecedent was reduced (Felser et al., 2009) have found no reliable effect of the inaccessible antecedent. This is consistent with recent findings showing that while sentences presented in isolation provide evidence for a syntax-based account of sentence processing, structural parsing mechanisms are influenced by discourse factors when sentences are placed in a more natural context (Yang et al., 2013).

In conclusion, our findings support a multiple constraint or cue-based retrieval approach to reflexive resolution whereby each potential antecedent is promoted by a variety of factors (both structural and discourse related), and while strong weighting is given to the structural constraint, non-structural cues or constraints (such as discourse prominence) can also affect online reflexive resolution. Furthermore, we suggest that behavioral measures may be influenced by the specific task participants are given and particularly that later occurring effects may reflect more conscious/controlled processes, as has also been reported in previous ERP research.

## Conflict of Interest Statement

The authors declare that the research was conducted in the absence of any commercial or financial relationships that could be construed as a potential conflict of interest.

## Footnotes

1 It should be noted that priming effects were only tested for at the point of the reflexive, not shortly after where effects have subsequently been found.

2 In both experiments numerical trends suggested an effect, but these were non-significant in the statistical analysis.

3 A further study reporting significant interference from an inaccessible antecedent in the processing of reflexives used eye-tracking during listening to investigate the interpretation of picture noun phrases (Runner et al., 2003). However, the authors concluded that reflexives in such contexts are in fact “logophors” and thus exempt from Binding Theory [see also Runner et al. (2006)].

4 The selected picture stimuli could be easily recognised, as shown by their mean “visual recognisability” score of 97% (SD: 6%, range: 80–100%).

5 To avoid gaze direction coding being influenced by coders’ expectations, coding was initially done “blind,” so that gaze direction was coded as being toward the top left, top right, bottom left, bottom right, center, or off-screen (i.e., participant blinking or not looking at screen), without the coder knowing the arrangement of the pictures in the visual display the participant was viewing. Gaze directions were then re-coded with reference to the visual display to show whether the participant was looking at the accessible antecedent, the inaccessible antecedent, the object, the distracter character, the center, or off-screen.

6 We thank a reviewer for pointing out these two subtly different interpretations.

7 Perhaps because the low salience of the reflexive in the naturalistic design meant that in a large number of trials participants did not look at any potential antecedent on hearing the reflexive, thus reducing the number of valid data points and leading to a low-power analysis.
