# Supplementary material for: Reflexive anaphor resolution in spoken language comprehension: structural constraints and beyond
Source: Front Psychol. 2014 Aug 19;5:904. doi: 10.3389/fpsyg.2014.00904 (PMC4137754; doi:10.3389/fpsyg.2014.00904)
Supplement: Supplementary file 1 [file Data_Sheet_1.DOCX]

# Appendix

Appendix A: Fixed effects from best fitting mixed-effects logistic regression models (only data from correctly answered trials analysed)

| Fixed Effects | Estimate | St.Error | *z* value | *p* value |
| --- | --- | --- | --- | --- |
| **Time Window 1: 200-600ms** | | | | |
| (Intercept) | 4.992 | 2.786 | 1.792 | 0.073 |
| Time | 41.726 | 7.140 | 5.844 | <.001* |
| Ant(Inacc.) | -23.378 | 5.569 | -4.198 | <.001* |
| Condition(Double-Match) | -2.846 | 2.840 | -1.002 | 0.316 |
| Time x Ant(Inacc.) | -65.102 | 14.745 | -4.415 | <.001* |
| Time x Condition(Double-Match) | -11.641 | 6.793 | -1.714 | 0.087 |
| Ant(Inacc.) x Condition(Double-Match) | 6.694 | 5.152 | 1.299 | 0.194 |
| Time x Ant(Inacc.) x Condition(Double-Match) | 31.792 | 10.455 | 3.041 | 0.002* |
| **Time Window 2: 600-1000ms** | | | | |
| (Intercept) | 10.411 | 1.429 | 7.285 | <.001* |
| Time | -3.621 | 4.799 | -0.755 | 0.450 |
| Ant(Inacc.) | -27.732 | 2.941 | -9.429 | <.001* |
| Condition(Double-Match) | 3.089 | 2.302 | 1.342 | 0.180 |
| Time x Ant(Inacc.) | 6.372 | 15.576 | 0.409 | 0.682 |
| Time x Condition(Double-Match) | 20.777 | 7.005 | 2.966 | 0.003* |
| Ant(Inacc.) x Condition(Double-Match) | -3.256 | 4.124 | -0.790 | 0.430 |
| Time x Ant(Inacc.) x Condition(Double-Match) | -39.849 | 18.122 | -2.199 | 0.028* |
| **Time Window 3: 1000-1400ms** | | | | |
| (Intercept) | 14.570 | 2.067 | 7.049 | <.001* |
| Time | -15.944 | 5.729 | -2.783 | 0.005* |
| Ant(Inacc.) | -44.342 | 5.478 | -8.094 | <.001* |
| Condition(Double-Match) | 1.984 | 3.156 | 0.628 | 0.530 |
| Time x Ant(Inacc.) | 62.577 | 17.439 | 3.588 | <.001* |
| Time x Condition(Double-Match) | -9.453 | 8.831 | -1.070 | 0.284 |
| Ant(Inacc.) x Condition(Double-Match) | -5.412 | 8.575 | -0.631 | 0.528 |
| Time x Ant(Inacc.) x Condition(Double-Match) | -39.850 | 22.241 | -1.792 | 0.073 |
| **Time Window 4: 1400-1800ms** | | | | |
| (Intercept) | -10.173 | 4.432 | -2.295 | 0.022* |
| Time | -21.193 | 8.959 | -2.366 | 0.018* |
| Ant(Inacc.) | -82.194 | 28.607 | -2.873 | 0.004* |
| Condition(Double-Match) | 28.226 | 5.046 | 5.594 | <.001* |
| Time x Ant(Inacc.) | -38.355 | 48.173 | -0.796 | 0.426 |
| Time x Condition(Double-Match) | 0.590 | 16.486 | 0.036 | 0.971 |
| Ant(Inacc.) x Condition(Double-Match) | -24.920 | 34.553 | -0.721 | 0.471 |
| Time x Ant(Inacc.) x Condition(Double-Match) | 84.467 | 60.952 | 1.386 | 0.166 |
| **Formula in R:**  DepVar ~ Time * Ant * Condition + (1 + Time * Ant * Condition \| Part) + (1 + Time * Ant \| Item) + (1 + Time * Ant \| Trial) | | | | |
